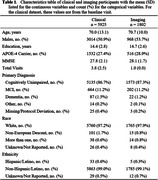# A Large Public Release of Clinical and Imaging Data from the Mayo Clinic Study of Aging

**DOI:** 10.1002/alz.091346

**Published:** 2025-01-09

**Authors:** Christopher G. Schwarz, Walter K. Kremers, Carl M. Prakaashana, Scott A. Przybelski, Luke R. Christenson, Josie M. Wiliams, Jeffrey L. Gunter, Matthew L. Senjem, Arvin Arani, Robert I. Reid, Mary M. Machulda, Julie A. Fields, Val J. Lowe, Kejal Kantarci, Jonathan Graff‐Radford, Prashanthi Vemuri, Ronald C. Petersen, David S. Knopman, Clifford R. Jack

**Affiliations:** ^1^ Mayo Clinic, Rochester, MN USA; ^2^ Department of Radiology, Mayo Clinic, Rochester, MN USA; ^3^ Department of Psychiatry and Psychology, Mayo Clinic, Rochester, MN USA

## Abstract

**Background:**

The Mayo Clinic Study of Aging (MCSA) is a longitudinal, population‐based study of residents of Olmsted County, Minnesota, USA. MCSA is releasing de‐identified clinical and imaging data on GAAIN.org to benefit the research community.

**Method:**

We included longitudinal clinical data from all participants of the Mayo Clinic Study of Aging 30‐90 years of age (average 70.0y) through the first 15 years of the study. Clinical data includes age, sex, self‐reported race/ethnicity, APoE4 allele status, cognitive impairment status (normal, MCI, dementia, or other), height, weight, blood pressure, multi‐domain neuropsychological scores, neuroimaging summary measurements, medications, cardiovascular risk factors, social activity, sleep, mood/anxiety, functional measures, and more.

For our initial release of imaging data, we selected the first imaging timepoint acquired on 3T GE MRI scanners and amyloid (PiB) PET during the same visit, from all available imaging participants 50‐90 years of age (average 70.7y). Currently, we included 3D T1‐weighted (MPRAGE) MRI (1x1x1.2mm^3^), T2‐weighted FLAIR MRI (0.85x0.85x3.6mm^3^), diffusion MRI (2.7mm resolution with 41 directions at b=1000 and 5 b=0), and numeric cortical global SUVR and Centiloid values from PiB PET scans at 40‐60 minutes post‐injection. All images are released in DICOM format and thoroughly de‐identified using CTP while ensuring preservation of necessary fields for analyses. T1‐weighted and T2‐FLAIR MRI were also de‐faced (identifiable face imagery was removed and replaced with an average) using mri_reface version 0.3.3 to further protect participants’ privacy, and all de‐faced images were visually inspected by a trained data scientist for quality assurance.

**Result:**

The first phase of our public data release includes clinical data from 5925 unique participants from the Mayo Clinic Study of Aging (MCSA), each ranging from 1‐12 visits at roughly 15‐month intervals, and three MRI sequences (T1‐w, T2‐w‐FLAIR, and diffusion) from 1802 unique participants. Demographic summaries are given in Table 1.

**Conclusion:**

This large, richly characterized, de‐identified clinical and imaging dataset is available to the research community at https://www.gaaindata.org/partner/MCSA, and it is suitable for a wide range of research uses. Additional data will be released over time, including de‐faced amyloid PET images.